# The effect of 1,25-dihydroxyvitamin D3 on liver damage, oxidative stress, and advanced glycation end products in experimental nonalcoholic- and alcoholic- fatty liver disease

**DOI:** 10.3906/sag-2007-289

**Published:** 2021-06-28

**Authors:** İlknur BİNGÜL, A. Fatih AYDIN, Canan KÜÇÜKGERGİN, Işın DOĞAN-EKİCİ, Semra DOĞRU-ABBASOĞLU, Müjdat UYSAL

**Affiliations:** 1 Department of Medical Biochemistry, İstanbul Medical Faculty, İstanbul University, İstanbul Turkey; 2 Department of Pathology, Acıbadem University Medical Faculty, İstanbul Turkey; 3 Tayyareci Nurettin Sokak, Bakırkoy, İstanbul Turkey

**Keywords:** Vitamin D, fructose, ethanol, oxidative stress, glycation end products

## Abstract

**Background/aim:**

Oxidative stress and advanced glycation end products (AGEs) formation are proposed as effective mechanisms in the pathogenesis of nonalcoholic fatty liver disease (NAFLD) and alcoholic liver disease (ALD). 1,25(OH)2D3 was proposed to have antioxidant, antiinflammatory and antiglycation properties. In this study, the effect of 1,25(OH)2D3 treatment on oxidative stress parameters and AGEs levels together with hepatic histopathology was investigated in high fructose (HFr) or ethanol (EtOH)-treated rats.

**Materials and methods:**

Rats were treated with fructose (30%) or ethanol (5-20%) in drinking water with and without 1,25(OH)2D3 treatment (5 µg/kg two times a week) for 8 weeks. Insulin resistance (IR), oxidative stress parameters, AGEs, triglyceride (TG), and hydroxyproline (Hyp) levels together with histopathology were investigated in the liver.

**Results:**

1,25(OH)2D3 decreased hepatic reactive oxygen species, lipid and protein oxidation products together with histopathological improvements in HFr- and EtOH-treated rats. 1,25(OH)2D3 treatment was observed to decrease significantly serum and hepatic AGEs in HFr group, and hepatic AGEs in EtOH group.

**Conclusion:**

Our results clearly show that 1,25(OH)2 D3 treatment may be useful in the alleviation of hepatic lesions by decreasing glycooxidant stress in both NAFLD and ALD models created by HFr- and EtOH-treated rats, respectively.

## 1. Introduction

Nonalcoholic fatty liver disease (NAFLD) is a common chronic liver disease. Steatosis is the first lesion in NAFLD. It is benign and reversible lesion. The presence of steatosis makes the liver susceptible to some factors such as oxidative stress, endotoxemia, inflammation and mitochondrial dysfunction. Thus, the development of steatosis to advanced lesions such as nonalcoholic steatohepatitis (NASH), fibrosis and cirrhosis is induced [1,2]. In addition, advanced glycation end products (AGEs) were proposed as a contributing factor in the pathogenesis of NAFLD [3,4]. Therefore, basic therapeutic strategies are directed to prevent the transformation of steatosis into more advanced lesions in the liver [1–4]. Since oxidative stress is considered as the main factor playing a role in this transformation, the effect of several antioxidants on hepatic lesions have been tested in experimental models of NAFLD [2,5].

There are two main form of Vitamin D (Vit D). They are Vit D2 (ergocalciferol) and Vit D3 (cholecalciferol). Vit D2 can be derived from the diet, but Vit D3 is synthesized from 7-dehydrocholesterol with the effect of ultraviolet light on the skin. Dietary or synthesized Vit D is metabolized with hydroxylation reactions. 25-hydroxyvitamin D [25(OH)D] is a product of the first hydroxylation reaction in the liver. This product is used as an indicator of Vit D stores. The second hydroxylation reaction occurs in the kidneys, resulting in the production of 1,25 dihydroxyvitamin D [1,25(OH)2D], which is the biologically active form of Vit D [6,7].

1,25(OH)2D functions by binding to receptors (Vit D receptor; VDR) located in the nucleus. Primary target tissues of Vit D are the bone, kidney, and intestines. However, these receptors are also available in several tissues such as immune and endocrine systems, muscles, brain and liver. Therefore, in addition to regulating bone homeostasis, Vit D may regulate several genes and influence immune system functions, cellular proliferation and differentiation, oxidative stress, protein glycation, inflammation, and apoptosis [6–9]. Therefore, Vit D has been reported to play an effective role in preventing many diseases such as diabetes mellitus, hypertension, cardiovascular diseases, autoimmune diseases, and cancer [6–9]. 

It has been proposed that low levels of Vit D may have an effective role in the development of insulin resistance (IR), metabolic syndrome (MS) and NAFLD [10,11].

The mechanisms underlying the association between Vit D and NAFLD has not been resolved yet [11,12]. Studies in dietary models of NAFLD/NASH such as chronic feeding of high fat- [13,14], western- [15] and methionine choline deficient (MCD)- [16,17] diets have shown that Vit D3 or 1,25(OH)2 D3 treatments may be effective in the prevention of the NAFLD formation and development by suppressing oxidative stress, the production of cytokines, apoptosis, steatosis, and fibrosis. Although the high-fructose (HFr) diet is one of good dietary models which is used to create metabolic syndrome and NAFLD/NASH in experimental animals [18], knowledge about the efficiency of 1,25(OH)2 D3 is limited in HFr-treated animals [19,20]. 

On the other hand, alcoholic liver disease (ALD) is a common chronic liver disease such as NAFLD. The liver is the main organ of alcohol metabolism. Several factors such as steatosis, increases in production of reactive oxygen species (ROS), accumulation of toxic acetaldehyde (AA), AA-induced AGEs formation, and mitochondrial damage play a role in alcohol-induced hepatotoxicity [3,21–24]. The mechanisms leading to the formation and progression of hepatic lesions in ALD are very similar to the mechanisms seen in NAFLD [3,23,24]. Studies investigating the association between ALD and Vit D have been obtained mostly from patients with ALD [25–27]. However, obtained data are controversial and there is no experimental study. 

Oxidative stress and AGEs formation are proposed as effective mechanisms in the pathogenesis of both NAFLD and ALD. In this study, the effect of 1,25(OH)2D3 treatment on oxidative stress parameters and AGEs levels together with hepatic histopathology was investigated in experimental NAFLD and ALD models created by HFr or ethanol (EtOH) administration, respectively.

## 2. Materials and methods

### 2.1. Chemicals

Fructose (Fr), ethanol (EtOH) and other chemicals were purchased from Sigma-Aldrich (Saint-Louise, MI, USA). 1,25(OH)2D3 (Ostriol, 2µg/mL) was donated by VEM ILAC San. A.S. (Istanbul, Turkey).

### 2.2. Animals 

Male Wistar rats (140-160 g) were provided from Aziz Sancar Experimental Medical Research Institute of Istanbul University. The animals were supplied with food and water ad libitum *.* They were kept in polypropylene cages (3–4 per cage) at 22 °C, with 12-h light and 12-h darkness. Total food and water intake was recorded daily. The experimental process used in this study was conducted according to Guidelines for the Animal Care and Use Committee of the University of İstanbul (approval no: March 29, 2018-2108/28). 

### 2.3. Diets and experimental design

Laboratory chow diet (containing 2300 IU vitamin D3/kg, PicoLab rodent diet 20) was purchased from LabDiet (St. Louis, MO, USA). All groups were fed on this diet during the 8-week experimental period. The dose and duration of the 1,25(OH)2D3 injection were determined according to the previous studies [13,17].

Rats were randomly assigned into five groups: a) Control group (n = 6): rats were given drinking water and injected with saline as the vehicle, b) High fructose group (HFr; n = 7): rats received fructose (30%; w/v, in drinking water), c) Ethanol group (EtOH; n = 7): rats were treated with EtOH in drinking water in increasing concentrations. They were treated with 5% (v/v) and 10% (v/v) EtOH in drinking water for the first and second weeks respectively, to ensure the adaptation of the rats to the ethanol. For the last 6 weeks, 20% (v/v) EtOH was administered, d) HFr + 1,25(OH)2D3 group (n = 7): rats were given Fr in drinking water and injected with 1,25(OH)2D3 (5µg/kg; twice a week) intraperitoneally, e) EtOH+ 1,25(OH)2D3 group (n = 7): rats received EtOH in drinking water and injected with 1,25(OH)2D3 (5µg/kg; twice a week) intraperitoneally. 

### 2.4. Samples

At the end of the experimental period, animals were exposured to overnight fasting and anesthetized with ketamine (35 mg/kg, i.p., Pfizer, USA) and xylazine HCl (15 mg/kg, i.p., Bioveta, Czech Republic). Blood was collected in dry tubes by cardiac puncture and serum was obtained by centrifugation. The livers were removed, and washed with ice-cold 0.9% NaCl and kept in ice. The liver index was calculated as liver weight/body weight × 100. Liver tissue was homogenized in ice-cold 0.15 M KCl (10%; w/v) and centrifuged at 600 g for 10 min at 4 °C to obtain the postnuclear fraction (PNF). The materials were stored at –80 °C until they were analyzed and biochemical analyses in the liver were performed in this fraction. 

### 2.5. Serum parameters

Serum fasting glucose, total cholesterol (TC) triglyceride (TG), calcium and inorganic phosphorus levels, and alanine aminotransferase (ALT) and aspartate aminotransferase (AST) activities were measured using a Cobas Integra 800 autoanalyzer (Roche Diagnostics, Mannheim, Germany). Serum 25(OH)D3 rRat 25-hydroxy vitamin D3; Abbkine Wuhan, China), insulin (rat insulin; Abbkine Wuhan, China), and Nε-(carboxymethyl) lysine (CML; rat N(6)-carboxymethyllysine; Abbkine Wuhan, China) levels were measured using ELISA kits in accordance with the manufacturers’ instructions. The homeostasis model known as the assessment (HOMA) index was used to evaluate insulin resistance (IR) and calculated using the formula: fasting insulin concentration (pmol/L) × fasting glucose concentration (mmol/L)/135 as previously reported [28]. High HOMA scores indicate IR (low insulin sensitivity). To determine advanced glycation end products (AGEs), serum samples were diluted with phosphate-buffered saline (PBS) pH 7.4, and fluorescence intensity was measured (λemission: 440 nm; λexcitation: 350 nm). Results were expressed as arbitrary units (RFU) [29].

### 2.6. Hepatic TC and TG levels 

Hepatic lipids were extracted with chloroform:methanol (2:1) [30] and hepatic TC and TG levels were assayed using kits provided by Biolabo Biochemistry and Coagulation (Maizy, France). Results were expressed in µmol/g tissue.

### 2.7. Hepatic hydroxyproline (Hyp) levels 

Liver tissues were minced to small pieces and homogenized in 10 volumes of PBS (0.01M, pH: 7.4) with a glass homogenizer on ice. To further break the cells, tissue homogenates were sonicated with an ultrasonic cell disrupter and then centrifuged at 5000 g for 5 min to get the supernatants. Hyp levels were evaluated using rat hydroxyproline (Bioassay Technology Laboratory, Shanghai, China) ELISA kits in accordance with the manufacturers’ instructions. Results were expressed as ng/mL homogenate. 

### 2.8. Hepatic reactive oxygen species (ROS) levels

ROS generation was determined by the method described by Wang and Joseph [31]. After excitation at 485 nm, the fluorescence emission of 2′,7′-dichlorofluorescein at 538 nm was recorded using a microplate fluorometer and luminometer (Fluoroskan Ascent FL, Thermo Scientific Inc, USA). Results were reported as relative fluorescence units (RFU).

### 2.9. Hepatic lipid peroxide levels

Hepatic lipid peroxidation was measured by the determination of thiobarbituric acid reactive substances (TBARS) and diene conjugate (DC) levels. TBARS levels were determined according to Buege and Aust [32]. The breakdown product of 1,1,3,3-tetraethoxypropane was used as a standard. Results were reported as pmol/mg protein. To determine DC levels, liver lipids were extracted in chloroform/metanol (2:1) and then redissolved in cyclohexane. Absorbances at 233 nm were recorded. Results were calculated using a molar extinction coefficient of 2.52 × 104 M−1cm−1. Results were reported as µmol/g tissue [32]. 

### 2.10. Hepatic protein carbonyl (PC) levels

PC levels were evaluated according to the method of Reznick and Packer [33] which is based on the measurement of protein hydrazones formed by the reaction between 2,4-dinitrophenylhydrazine and protein carbonyl groups. Results were calculated from the maximum absorbance (360 nm) using a molar extinction coefficient of 22,000 M−1cm−1 and reported as nmol carbonyl per mg protein.

### 2.11. Hepatic advanced oxidation products of protein (AOPP) levels

AOPP levels were measured spectrophotometrically at 340 nm according to the method of Hanasand et al [34]. Results were expressed as nmol/chloramine-T equivalent/mg protein.

### 2.12. Hepatic AGE levels 

AGEs levels were determined spectrofluometrically. For this reason, liver homogenates were diluted with phosphate-buffered saline (PBS) pH 7.4 and the fluorescence intensity (λemission: 440 nm; λexcitation: 350 nm) was determined [29]. Results were expressed in arbitrary units (RFU).

### 2.13. Hepatic ferric reducing anti‐oxidant power (FRAP) levels 

FRAP assay was used for the determination of antioxidant power in liver. A ferric-tripyridyltriazine complex is reduced to the ferrous form by the action of electron donating antioxidants present in liver tissue. The reaction is monitored by measuring the change in absorption at 593 nm. Results were reported as nmol/mg protein [35].

### 2.14. Hepatic glutathione (GSH) levels

GSH levels were measured by using 5,5-dithiobis-(2-nitrobenzoate) at 412 nm. Results were expressed as nmol/mg protein [36]. 

### 2.15. Protein levels

Protein levels were determined spectrophotometrically using bicinchoninic acid [37].

### 2.16. Histopathologic examination

Livers were fixed in 10% buffered formalin, embedded in paraffin, sectioned and stained with hematoxylin and eosin (H&E) for histologic examinations. Masson’s trichrome (MTC) staining was also performed to show reticulin fibers of fibrotic areas. Steatosis, liver damage, and fibrosis scores were made according to the protocol proposed by Goodman [38], as previously described in detail in our previous study [39].

### 2.17. Statistical analysis

Statistical analysis was evaluated by using the Statistical Package for The Social Sciences program v: 21.0 (SPSS Inc., Chicago, IL, USA) program. Data distributions and test of normality were investigated by Kolmogorov–Smirnov test. All variables were expressed as mean ± standard deviation (SD). The significance between groups for parameters with normal distribution was compared by using one-way ANOVA test. Homogeneity of variances was evaluated with Levene test. In cases of significant difference between groups, two-way post-hoc comparisons were performed with the Tukey test. The significance between groups for parameters without normal distribution was compared by using the Kruskal–Wallis test. In case of significant difference between groups two-way comparisons were completed by using Bonferroni-corrected Mann–Whitney U test. In all cases, a difference was considered significant when p < 0.05.

## 3. Results

### 3.1. Body weight, liver weight, and liver index 

Daily water intake did not change, however food intake decreased in HFr-rats. Although final body weight did not change, liver weight and liver index elevated. Final body weight and food intake decreased due to 1,25(OH)2D3 treatment in HFr rats. Daily food intake decreased, but water intake remained unchanged in EtOH-treated rats. There was a significant decrease in final body weight. Liver weight and liver index remained unchanged in EtOH group. There were no changes in these parameters due to 1,25(OH)2D3 treatment in EtOH-treated rats. When HFr and EtOH groups were compared, daily food intake was statistically lower, but water intake, liver weight and liver index were higher in HFr group (Table 1).

**Table 1:** The effect of 1,25(OH)2D3 treatment on final daily food and water intakes, body weights, liver weight and liver index* values in high fructose (HFr)- and ethanol (EtOH)-treated rats (Mean ± SD).

	Control(n = 6)	HFr(n = 7)	EtOH(n = 7)	HFr+ 1,25(OH)2D3(n = 7)	EtOH+ 1,25(OH)2D3(n = 7)
Food intake (g/day)	24.8 ± 0.73	11.0 ± 0.50a	15.5 ± 1.38a,b	12.4 ± 0.67a,c	15.2 ± 1.51a
Water intake (mL/day)	35.6 ± 2.05	39.0 ± 3.58	28.5 ± 3.74b	39.2 ± 7.22	28.9 ± 3.23
Final body weight (g)	299.5 ± 16.2	312.1 ± 31.6	276.2 ± 40.9	285.0 ± 12.6	263.0 ± 14.7a
Liver weight (g)	8.25 ± 0.93	10.3 ± 1.47a	7.69 ± 1.48b	9.64 ± 0.99	7.05 ± 0.67
Liver index (%)	2.75 ± 0.22	3.29 ± 0.27a	2.78 ± 0.26b	3.38 ± 0.30a	2.69 ± 0.28

### 3.2. Serum 25(OH)D3, calcium, and inorganic phosphorus levels 

There were no changes in serum 25(OH)D3, calcium and inorganic phosphorus levels among groups (Table 2).

**Table 2:** The effect of 1,25(OH)2D3 treatment on some biochemical parameters in serum of high fructose (HFr) and chronic ethanol (EtOH)-treated rats. (Mean ± SD).

	Control(n = 6)	HFr(n = 7)	EtOH(n = 7)	HFr+ 1,25(OH)2D3(n = 7)	EtOH+ 1,25(OH)2D3(n = 7)
25(OH)D3 (ng/mL)	28.4 ± 3.09	25.3 ± 5.88	27.2 ± 6.59	26.5 ± 1.42	26.7 ± 4.90
Calcium (mmol/L)	2.42 ± 0.17	2.36 ± 0.12	2.61 ± 0.20	2.59 ± 0.18	2.56 ± 0.18
Phosphorus (mmol/L)	2.55 ± 0.19	2.31 ± 0.16	2.48 ± 0.24	2.26 ± 0.29	2.26 ± 0.25
Glucose (mmol/L)	7.54 ± 1.44	12.2 ± 1.09a	6.51 ± 1.91b	11.3 ± 1.35a	6.66 ± 1.15
Insulin (pmol/L)	29.3 ± 4.38	24.5 ± 2.01	24.5 ± 11.5	21.9 ± 4.88	23.9 ± 7.26
HOMA	1.62 ± 0.33	2.22 ± 0.24a	1.22 ± 0.75b	1.83 ± 0.48	1.16 ± 0.36
TC (mmol/L)	1.67 ± 0.19	1.53 ± 0.15	1.62 ± 0.28	1.48 ± 0.22	1.70 ± 0.19
TG (mmol/L)	0.48 ± 0.09	1.14 ± 0.27a	0.90 ± 0.26a	0.89 ± 0.22a	0.82 ± 0.25

ap < 0.05 as compared to control; bp < 0.05 HFr vs. EtOH.

### 3.3. Glucose, insulin, HOMA (IR), total cholesterol (TC), and triglyceride (TG) levels

Glucose, TG, and HOMA levels increased, but insulin and TC levels remained unchanged In HFr rats. There were no changes in these parameters between HFr and 1,25(OH)2D3-treated HFr rats. Serum glucose, insulin, TC, and HOMA levels did not change, but serum TG levels increased in EtOH-treated rats. 1,25(OH)2D3-treatment did not affect these parameters in EtOH-treated rats (Table 2).

### 3.4. Serum ALT and AST activities

**Figure 1:**
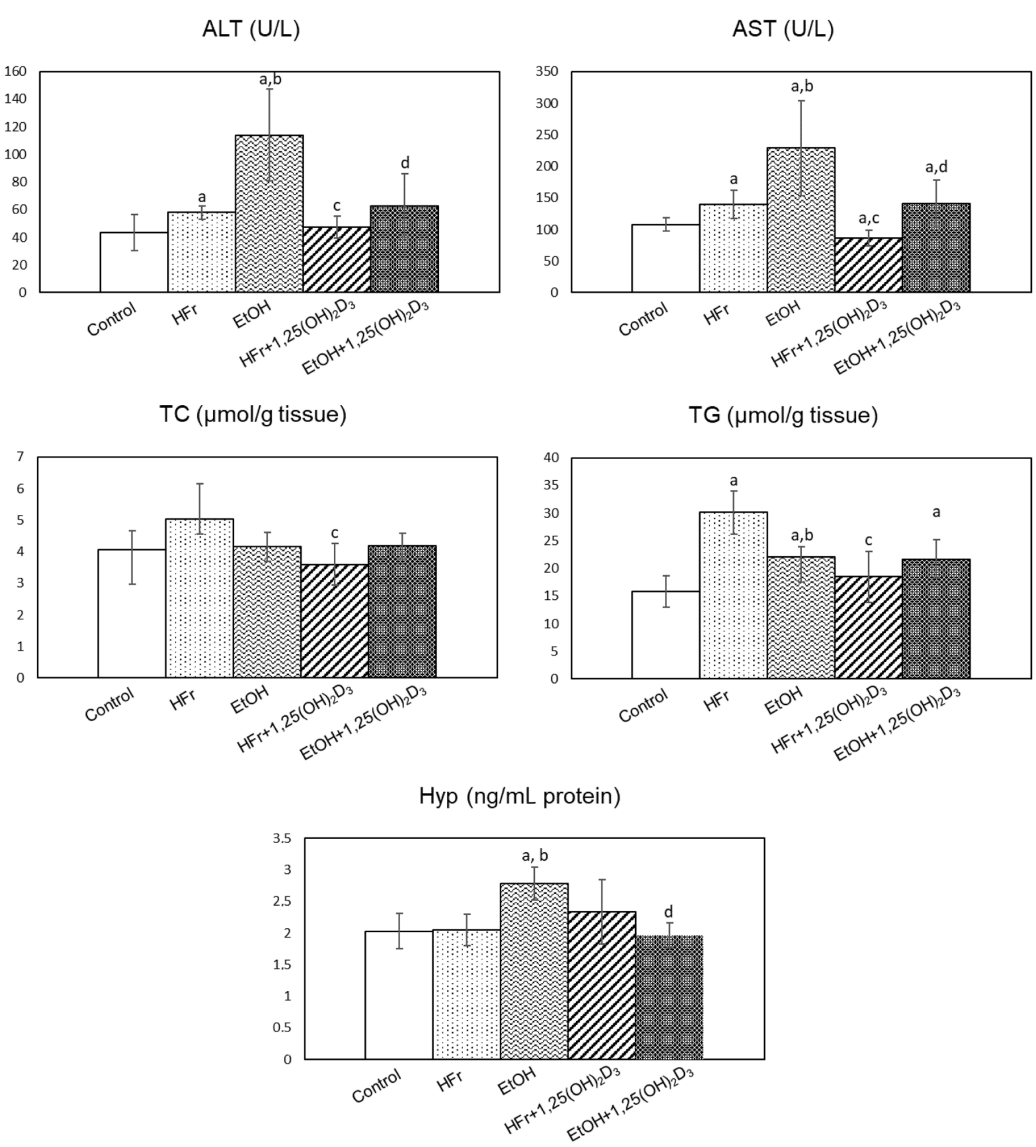
The effect of 1,25(OH)2D3 treatment on serum alanine aminotransferase (ALT) and aspartate aminotransferase (AST) activities, and hepatic total cholesterol (TC), triglyceride (TG), and hydroxyproline (Hyp) levels in high fructose (HFr)- and chronic ethanol (EtOH)-treated rats (Mean   SD). ap < 0.05 as compared to control; bp < 0.05 HFr vs. EtOH; cp < 0.05 HFr vs. HFr + 1,25(OH)2D3; dp < 0.05 EtOH vs. EtOH+1,25(OH)2D3.

Serum ALT and AST activities were detected to increase in HFr- and EtOH-treated groups. These activities were observed to be higher in EtOH group than HFr group. 1,25(OH)2D3-treatment lowered ALT and AST activities significantly in both groups (Figure 1).

### 3.5. Liver TC and TG levels

Liver TG levels increased 1.9 and 1.4 folds in HFr and EtOH groups, respectively. High levels of TG diminished due to treatment in HFr group, but these levels remained unchanged in EtOH group (Figure 1). There were no significant changes in hepatic TC levels in HFr- and EtOH-treated groups. However, 1,25(OH)2D3 treatment resulted in significant decreases in TC levels in the liver of HFr-rats. 

### 3.6. Liver Hyp levels 

Hepatic Hyp levels did not alter in HFr and 1,25(OH)2D3 -treated HFr groups as compared to controls. However, significant increases in hepatic Hyp levels were detected in EtOH group. These high levels diminished significantly due to 1,25(OH)2D3 treatment. (Figure 1). 

### 3.7. Liver ROS, TBARS, DC, and PC levels 

**Figure 2:**
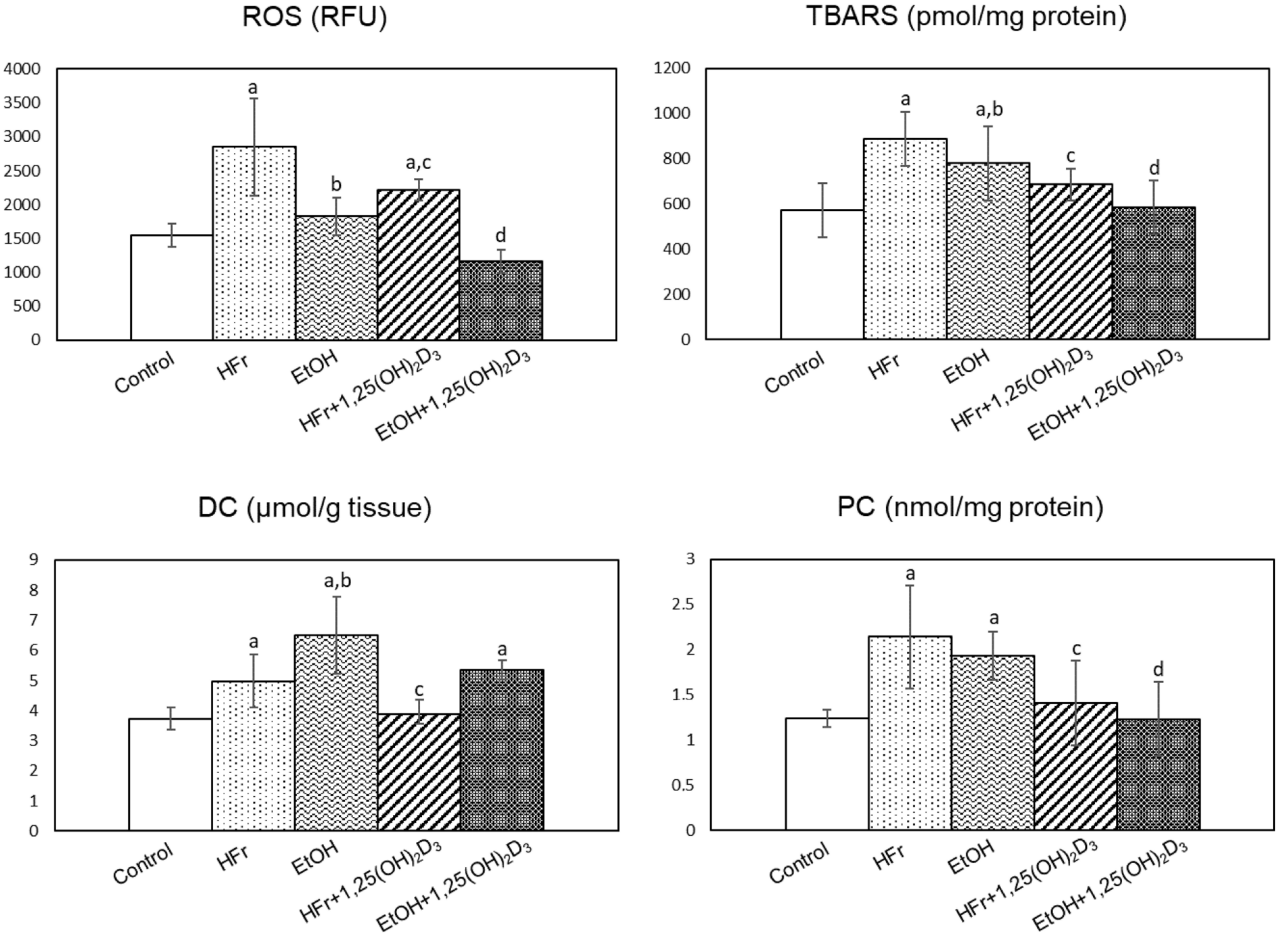
The effect of 1,25(OH)2D3 treatment on hepatic reactive oxygen species (ROS), thiobarbituric acid reactive substances (TBARS), diene conjugates (DC), and protein carbonyl (PC) levels in high fructose (HFr)- and chronic ethanol (EtOH)-treated rats (Mean   SD). ap < 0.05 as compared to control; bp <0.05 HFr vs/ EtOH; cp < 0.05 HFr vs. HFr + 1,25(OH)2D3; dp < 0.05 EtOH vs. EtOH + 1,25(OH)2D3.

ROS (84.4%), TBARS (54.6%), DC (33.2%), and PC (72.6%) levels were also significantly higher in HFr-rats than controls. 1,25(OH)2D3 treatment resulted in significant decreases in ROS (22.1%), TBARS (22.5%), DC (21.9%), and PC (34.1%) levels in HFr rats (Figure 2). Significant increases of TBARS (36.1%), DC (74.3%), and PC (55.6%), but not ROS (18.3%) levels were detected in EtOH-treated rats. ROS (36.1%), TBARS (25.0%), DC (17.8%; not significant), and PC (36.3%) levels decreased due to 1,25(OH)2D3 treatment in EtOH group (Figure 2).

### 3.8. Liver FRAP and GSH levels 

Liver FRAP (18.4%) and GSH (18.8%) levels tended to decrease in HFr-treated rats, but these decreases were not significant. These levels did not alter due to 1,25 (OH)2D3 treatment. Although there was no change in these parameters in EtOH group, FRAP (23.9%) and GSH (25.1%) levels increased due to 1,25 (OH)2D3 injection in EtOH-treated rats. However, these increases were also not significant (Table 3).

**Table 3:** The effect of 1,25(OH)2D3 treatment on hepatic ferric reducing antioxidant power (FRAP), glutathione (GSH), advanced oxidized protein products (AOPP), and advanced glycation end products (AGE) levels as well as serum AGE and carboxymethyllysine (CML) levels in high fructose (HFr)- and chronic ethanol (EtOH)-treated rats (Mean ± SD).

	Control(n = 6)	HFr(n = 7)	EtOH(n = 7)	HFr+ 1,25(OH)2D3(n = 7)	EtOH+ 1,25(OH)2D3(n = 7)
Hepatic FRAP (nmol/mg protein)	80.4 ± 11.9	65.6 ± 12.5	68.1 ± 13.4	63.8 ± 11.8	84.4 ± 11.4
Hepatic GSH (nmol/mg protein)	23.9 ± 3.02	19.4 ± 4.03	21.5 ± 5.17	19.5 ± 4.99	26.9 ± 5.70
Hepatic AOPP (nmol/mg protein)	22.8 ± 0.96	28.7 ± 3.02a	28.5 ± 3.13a	25.5 ± 2.35a	26.0 ± 4.70
Hepatic AGE (Rfu)	351.3 ± 18.2	549.6 ± 22.8a	524.5 ± 62.2a	347.4 ± 38.5c	330.4 ± 23.5d
Serum AGE (Rfu)	186.3 ± 15.5	250.6 ± 29.8a	227.8 ± 16.0a	163.7 ± 25.2c	221.8 ± 30.2
Serum CML (µg/L)	12.4 ± 1.75	17.3 ± 3.00a	13.3 ± 2.82b	12.9 ± 2.48c	12.6 ± 1.75

ap < 0.05 as compared to control; bp < 0.05 HFr vs. EtOH; cp < 0.05HFr vs. HFr + 1,25(OH)2D3.dp < 0.05 EtOH vs. EtOH+1,25(OH)2D3.

### 3.9. Hepatic AOPP and AGE levels, and serum AGE and CML levels 

Hepatic AOPP (25.8%) and AGE (49.3%) levels as well as serum AGE (22.3%) and CML (40.3%) levels were higher in HFr group as compared to controls. 1,25(OH)2D3 treatment was observed to decrease significantly serum AGE (28.1%) and CML (25.5%) and hepatic AGE (33.8%) levels. EtOH treatment elevated hepatic AOPP (25.0%), serum (34.5%) and hepatic (56.4%) AGE levels but not serum CML levels. Only hepatic AGE (39.9%) levels diminished following 1,25(OH)2D3 treatment in EtOH group (Table 3).

### 3.10. Histopathological results

**Figure 3:**
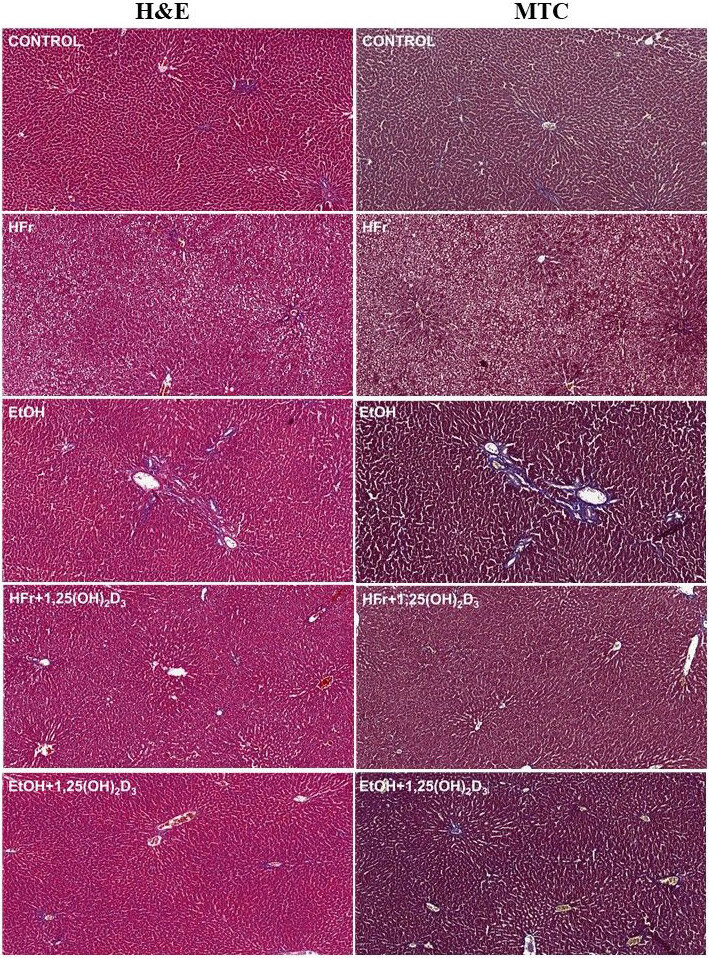
Hematoxylin and eosin (H&E) and Masson’s trichrome (MTC) staining of liver sections from high fructose (HFr)- and chronic ethanol (EtOH) groups treated by 1,25(OH)2D3. The control group showed normal hepatic architecture with central vein and radiating hepatic cords. HFr group exhibited marked microvesicular steatosis without fibrous expansion or fibrosis. EtOH group showed microvesicular steatosis not exceeding 5% and fibrotic changes. In 4 of the 7 rats, mild portal and periportal fibrous expansion, and in 3 of the 7 rats, fibrous expansion of most portal areas with occasional portal to portal (P-P) bridging was observed. HFr + 1,25(OH)2D3 group showed a decrease in steatosis and no fibrosis was detected when compared to HFr group. There was neither microvesicular steatosis nor hepatocyte ballooning in EtOH + 1,25(OH)2D3 group, but an improvement in fibrotic changes was observed as compared to the EtOH group.

Results of histopathological examination in H&E and MTC staining liver pieces were shown in Figure 3 and Table 4. Steatosis and hepatocyte ballooning scores were observed to increase in HFr rats, but there were no changes in fibrosis score. 1,25(OH)2D3 treatment significantly decreased the steatosis and hepatocyte ballooning scores in HFr-rats. Microvesicular steatosis not exceeding 5% and significant increase in fibrosis score were observed in EtOH-treated rats. There was no microvesicular steatosis and significant decreases in fibrosis score in 1,25(OH)2D3 treated- ETOH group.

**Table 4:** Mean scores of steatosis, ballooning, and fibrosis in high fructose (HFr) and ethanol (EtOH) groups with and without 1,25(OH)2D3 treatment (Mean ± SD).

	Control(n = 6)	HFr(n = 7)	EtOH(n = 7)	HFr+ 1,25(OH)2D3(n = 7)	EtOH+ 1,25(OH)2D3(n = 7)
Steatosis	0	3.00 ± 0.00a	0b	1.42 ± 0.79a,c	0
Ballooning	0	2.00 ± 0.00a	0b	1.28 ± 0.49a,c	0
Fibrosis	0	0	1.86 ± 1.07a,b	0	0.43 ± 0.53d

ap < 0.05 as compared to control; bp < 0.05 HFr vs. EtOH; cp < 0.05HFr vs. HFr + 1,25(OH)2D3.dp < 0.05 EtOH vs. EtOH + 1,25(OH)2D3.

## 4. Discussion

Fructose, a highly lipogenic nutrient is primarily metabolized in liver. HFr diet stimulates hepatic lipogenesis and causes IR and NAFLD. Oxidative stress and inflammation play an important role in the pathogenesis of HFr-induced metabolic changes and hepatic lesions [40]. In addition, increases in nonenzymatic protein glycation led to the formation of AGEs, which also play a role in HFr-induced toxicity [41]. AGEs affect structures and functions of proteins and cause further increases in oxidative stress and inflammation by interacting with their receptors [42]. Indeed, HFr diet application was reported to result in increased lipid and protein oxidation products, and decreased antioxidant parameters together with increased cytokines and AGEs levels in serum and liver [28, 43−47]. This application also caused hepatic lesions such as fatty liver, ballooning and lobular inflammation [28, 43−47]. However, some factors such as Fr content of diet, application time, and animal species may influence HFr-induced changes in animals.

In this study, rats received 30% fructose containing drinking water for 8 weeks as previously reported [46,47]. Increases in serum ALT and AST activities and marked microvesicular steatosis and hepatocyte ballooning were seen in the liver of HFr rats. These findings indicate that the hepatic lesions were produced successfully in NAFLD/NASH model. This diet also caused hyperglycemia, IR, and hepatic oxidative stress. 

As it is known, increases in ROS levels result in oxidative damage in lipid, protein and nucleic acids. TBARS and DC levels are lipid oxidation products. However, PC and AOPP levels are indicators of protein oxidation. In this study, ROS, TBARS, DC, PC, and AOPP levels were observed to increase in the liver of HFr rats. Under these conditions, hepatic FRAP, an indicator of antioxidant power, and GSH levels tended to decrease. This situation may reflect an insufficiency in antioxidant potential in the liver of HFr rats.

AGEs are heterogenous products and can be classified as fluorescent and nonfluorescent AGEs. Fluorescent spectroscopy is a valuable method for the determination of AGEs, but nonfluorescent AGEs such as CML cannot be measured using this method [42]. Moreover, AOPP, having some homologies with AGEs, reflects protein glycooxidation. Plasma AOPP levels were detected to correlate with plasma pentosidine and dityrosine levels which are indicators of protein glycation and oxidative protein damage, respectively [48]. In our study, increases in hepatic AGEs and AOPP as well as serum AGEs and CML levels indicate the presence of elevated HFr-induced protein glycation. 

ALD is one of causes of chronic liver disease. Chronic EtOH consumption can induce steatosis and advanced lesions such as alcoholic steatohepatitis, fibrosis and cirrhosis. Experimental evidence showed that chronic EtOH treatment resulted in significantly increased lipogenesis, ROS-induced oxidative stress and cytokine levels in the liver together with histopathological findings such as steatosis, inflammation, and fibrosis [49–53]. EtOH application causes increases in hepatic AA levels. AA accumulation was reported to induce the generation of AA-AGEs and play a role in the pathogenesis of ALD [3,54].

In this study, EtOH-induced liver injury was mediated by feeding rats with EtOH in drinking water for 8 weeks in increasing (5%−20%, v/v) concentrations, as previously reported [49,50,53]. This application caused significant increases in ALT and AST activities. Moreover, increased liver TG and Hyp levels together with microvesicular steatosis and fibrotic changes were detected in EtOH-treated rats. Lipid and protein oxidation products and AGEs levels also elevated as previously reported [49−54].

Information related to the effect of Vit D3 or 1,25(OH)2 D3 applications on HFr or EtOH-induced metabolic and hepatic changes is limited. Hepatic histopathology and oxidative stress parameters were reported not to change in Vit D3- [19,20] or 1,25(OH)2 D3 [55,56]-treated normal rodents. However, in rats that received 20% Fr in drinking water for 6 weeks, Vit D3 treatment was detected to improve some HFr-induced metabolic disturbances such as IR, hyperglycemia, and dislipidemia [19]. Vit D3 treatment was also observed to improve hepatic lesions such as steatosis and fibrosis and decrease elevated IR and expressions of genes of lipogenesis and inflammation in the liver of mice fed on HFr [20]. However, there is no study investigating the effect of Vit D3 or 1,25(OH)2 D3 applications on glycooxidant stress in HFr rats. Likewise, the subject has not been investigated in experimental animals treated with chronic ethanol.It has only been recently reported that Vit D deficiency may aggravate hepatic oxidative stress and inflammation in EtOH-treated rats [57].

Vit D3 and 1,25(OH)2D3 have antioxidant and antiinflammatory properties [8,58]. It has been suggested that the antioxidant property of Vit D is based on its structural similarity with the cholesterol. In addition, Vit D induces the expression of several molecules involved in the antioxidant system including GSH, glutathione peroxidase and superoxide dismutase [58,59]. Moreover, Vit D3 and 1,25(OH)2D3 have been reported to prevent glycation of proteins (60) and inhibit AGE-mediated complications by modifying AGE-RAGE system [9]. In this study, the main goal was to investigate the effect of 1,25(OH)2D3 treatment on oxidative stress parameters and AGEs levels together with hepatic histopathology in HFr- or EtOH-treated rats. According to our results obtained from NAFLD and ALD models created by HFr and EtOH treatment in rats, IR and higher hepatic lipid accumulation was detected in HFr group, whereas higher serum ALT and AST activities and fibrotic changes in the liver in EtOH group. There was no significant difference in glycooxidative stress parameters between the two groups. Under these conditions, 1,25(OH)2D3 treatment diminished hepatic ROS formation, lipid and protein oxidation products and inflammation in the liver together with histopathological improvements in the liver in HFr- and EtOH-treated rats. In addition, in EtOH- and especially HFr-treated rats, significant decreases in protein glycation products were observed due to 1,25(OH)2D3 treatment.

In conclusion, these results clearly show that 1,25(OH)2 D3 treatment may be useful in the alleviation of hepatic lesions by decreasing glycooxidant stress in both NAFLD and ALD models created by HFr- and ETOH-treated rats, respectively.
